# Commensal–Pathogen Interactions along the Human Nasal Passages

**DOI:** 10.1371/journal.ppat.1005633

**Published:** 2016-07-07

**Authors:** Silvio D. Brugger, Lindsey Bomar, Katherine P. Lemon

**Affiliations:** 1 Department of Microbiology, The Forsyth Institute, Cambridge, Massachusetts, United States of America; 2 Department of Oral Medicine, Infection and Immunity, Harvard School of Dental Medicine, Boston, Massachusetts, United States of America; 3 Division of Infectious Diseases, Boston Children’s Hospital, Harvard Medical School, Boston, Massachusetts, United States of America; Geisel School of Medicine at Dartmouth, UNITED STATES

## Why Study Nasal Microbiota?

**Fig 1:**
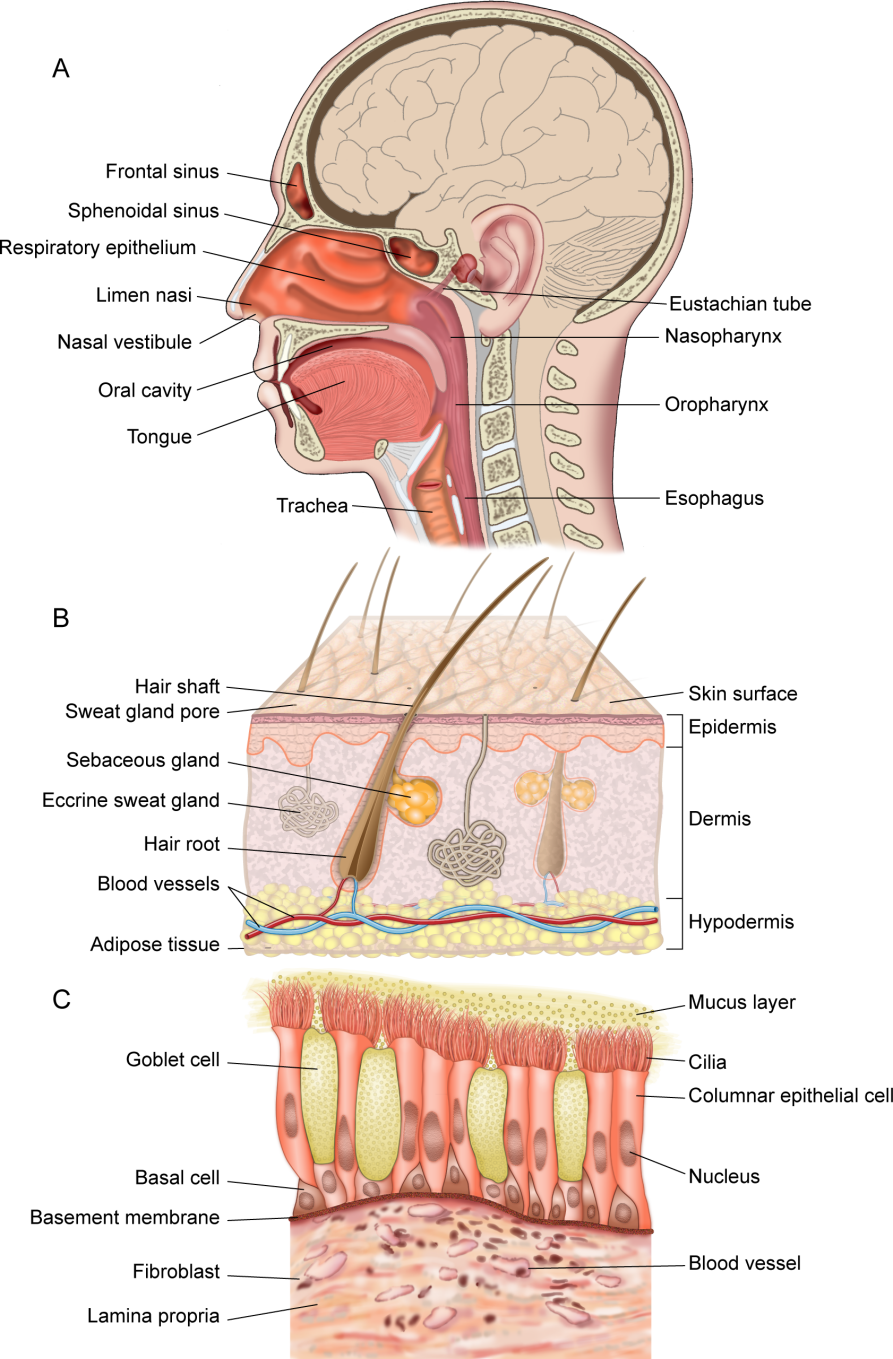
The human nasal passages. As reviewed in [5], the nostrils (anterior nares) are the entrance into the (A) human nasal passages (sagittal section) and open onto the skin-covered surface of the nasal vestibule, an acidic environment that contains sweat and sebaceous glands. (B) Cross section of the nostril skin. Moving posterior (A), the limen nasi marks the transition from the posterior region of the nasal vestibules to a mucosal surface, which contains mucin-secreting goblet cells and where the pH begins to steadily increase, reaching neutrality before the nasal cavity ends in the nasopharynx, the top of the back of the throat. Respiratory epithelial cells, including cilia that beat towards the esophagus, line the posterior segment of the nasal cavity and the nasopharynx. (C) Cross section of the mucosal surface.

Bacterial species that commonly reside on surfaces of the human nasal passages (Fig 1) interact with the host along a continuum from beneficial to harmful, i.e., from mutualist to commensal to pathogen. Likewise, the host responds along a continuum from tolerance to damage [1]. In fact, a small number of bacterial species that are prevalent and often abundant members of the nasal microbiota are important human pathogens, e.g., *Staphylococcus aureus* and *Streptococcus pneumoniae*. In the United States alone, *S*. *pneumoniae* contributes to ~20,000 deaths [2] and methicillin-resistant strains of *S*. *aureus* (MRSA) contribute to ~10,000 deaths annually [3]. Despite this significant mortality, most *S*. *aureus* and *S*. *pneumoniae* interactions with humans are harmless and do not result in disease, i.e., are commensal. However, benign colonization can be the starting point for disease, host–host transmission, and selection for new microbial traits. This duality of behavior from commensal to pathogen has led to the term pathobiont [4]. The factors that shift the behavior of pathobionts from a commensal to a pathogenic state remain to be identified. However, the interplay of pathobionts with other members of the microbiota might be one such factor. The combination of cultivation and 16S rRNA gene-based approaches has revealed new insights into this possibility and has sparked renewed interest in determining the molecular mechanisms of commensal–pathobiont interactions in the nasal microbiota. One goal of these efforts is to identify potentially beneficial bacteria (mutualists) that might either exclude pathobionts through colonization resistance or shift the behavior of colonizing pathobionts towards commensalism.

The feasibility of this goal is supported by two studies in adult Danish twins. Data from a large study of 617 twin pairs indicate that host genetics play a limited role in determining *S*. *aureus* nostril colonization and suggest a larger role for environmental factors, which could include the microbiota [6]. In a follow-up study of 46 monozygotic and 43 dizygotic twin pairs, the bacterial composition of the nasal microbiota is also predominantly an environmentally derived phenotype with host genetics playing a minor role in composition, but a larger role in determining bacterial density on nasal surfaces [7]. These studies, along with evidence that nasal microbiota composition changes over time, including seasonal variation [8–13], support the hypothesis that nasal microbiota composition could be altered for therapeutic benefit [7,14]. This hypothesis is bolstered by reports of negative correlations in colonization between key pathobionts (e.g., *S*. *pneumoniae* and *S*. *aureus*) and select benign commensals and of alterations in microbiota composition in disease states, e.g., middle ear infections (otitis media) [7,15–26]. Such studies further highlight the need to understand the role and function of the bacterial species that commonly reside in the human nasal passages and commensal–pathobiont interactions.

**Fig 2:**
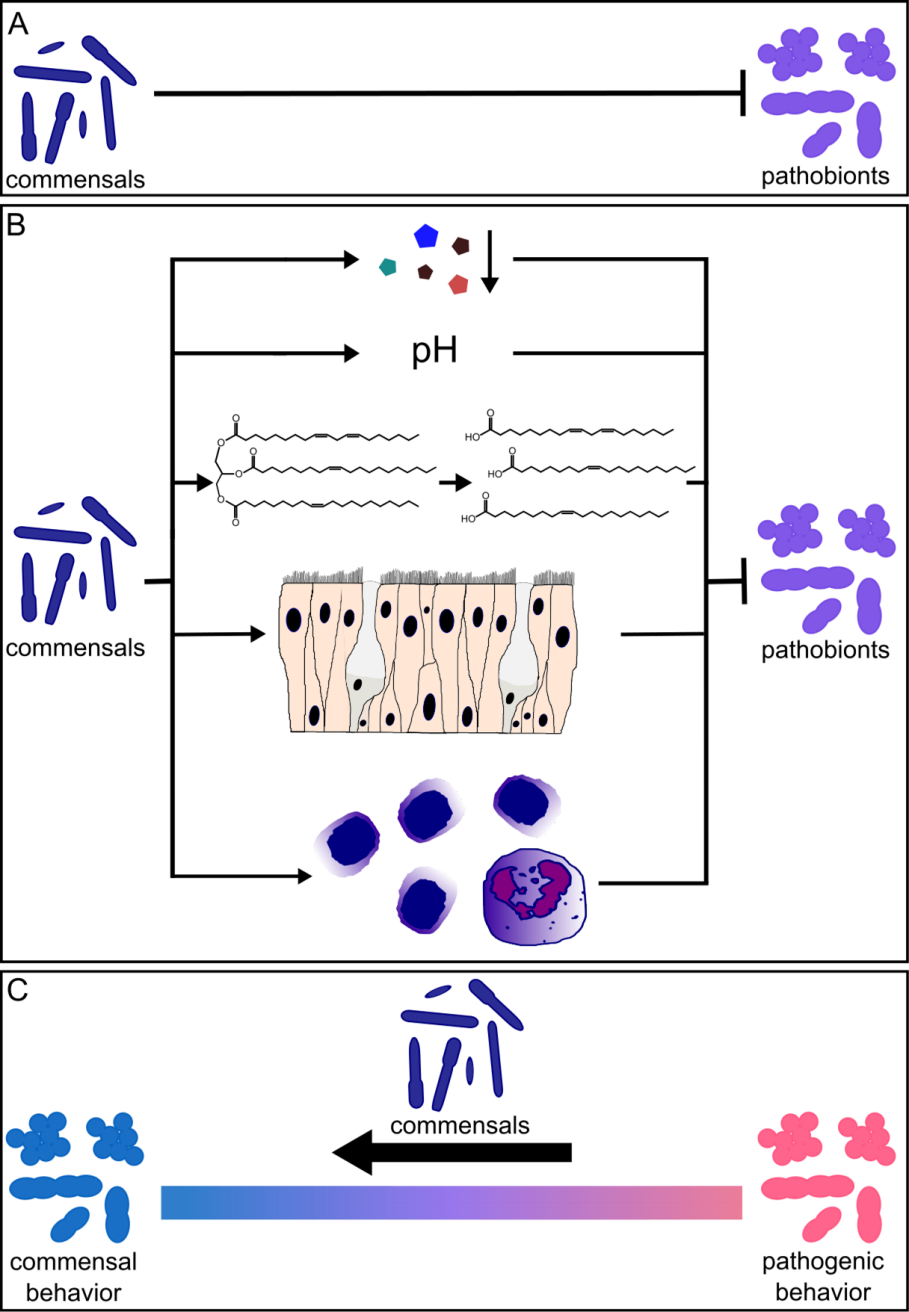
Commensal–pathobiont interactions that are beneficial to the host. Commensal bacteria can impact pathobionts in a manner beneficial to the host via (A) **direct inhibition**, e.g., production of antimicrobials; (B) **indirect inhibition**, e.g., competition for nutrients, modification of the habitat via acidification of environmental pH, alteration of host compounds or secretion of toxic metabolite(s), promotion of host epithelial barrier function, or stimulation of the host immune system; or by (C) **behavior modification,** e.g., shifting pathogens towards commensalism.

As recently reviewed for gut microbiota [27] and illustrated in Fig 2, beneficial nasal bacteria might impact pathobionts by inhibiting colonization and proliferation (colonization resistance) via (A) direct or (B) indirect mechanisms or by (C) shifting pathogen behavior towards commensalism and away from pathogenesis (Fig 2). Here we review some recent advances in determining the molecular mechanisms of commensal–pathobiont interactions among bacterial members of the microbiota of the nasal passages.

## What Is Known about the Composition of the Bacterial Microbiota of the Nasal Passages?

The human nasal passages and nasopharynx (Fig 1) host a distinctive bacterial community that is increasingly well characterized via culture-independent 16S rRNA gene surveys of different age groups in health and disease (e.g., as reviewed in [28]). In prepubertal children, members of the phyla Firmicutes, Proteobacteria, Actinobacteria, and Bacteroidetes commonly colonize the nostrils and nasopharynx with the latter two exhibiting lower relative abundance on average than the other two [8,9,12,13,17,18,20,23,29,30]. Of the Actinobacteria, *Corynebacterium* is typically the dominant genus detected [8,12,23,29]. A clear shift in the nostril microbiota occurs during puberty [29] and persists in the majority of healthy adults under age 65 years; it becomes dominated by Actinobacteria, in particular the genera *Corynebacterium* and *Propionibacterium*, and Firmicutes, in particular the genus *Staphylococcus* [7,11,16,19,21,24,25,29,31–35]. In spite of transitions in the type of epithelial surface from skin to respiratory epithelium (Fig 1), a high similarity exists in the bacterial microbiota along the length of the nasal passages [19,33]; however, the nasal cavity is reported to host a more diverse bacterial community [19]. Among the common members of the nasal microbiota in both children and adults are known pathobionts.

## What Are the Common Nasal Pathobionts?

The primary middle ear and respiratory bacterial pathogens, *S*. *pneumoniae*, *Haemophilus influenzae*, *and Moraxella catarrhalis*, commonly colonize the nasal passages of healthy children, with *S*. *aureu*s usually present less often. In contrast, *S*. *aureus* colonization of the nasal passages is much more common in adults, whereas *S*. *pneumoniae* and *H*. *influenzae* are less common. We favor the term pathobiont for these species because pathobionts are by definition commensal members of the host’s microbiota, whereas opportunistic pathogens may be commensal, environmental, or zoonotic in origin. In addition, these nasal pathobionts can cause infection in healthy hosts, e.g., healthy children and adolescents, whereas opportunistic pathogens generally only infect hosts who are compromised in some manner, e.g., decreased immune function or a significant breakdown in barrier function, such as with traumatic open wounds. (Although of great interest, the research on pathobiont–pathobiont interactions in the upper respiratory tract is beyond the scope of this review.) Recent upper respiratory tract cross-sectional studies that combine culture-based detection of pathobionts with high-throughput-sequencing characterization of microbiota in adults and children reveal inverse correlations between levels of *S*. *aureus* or *S*. *pneumoniae* and specific commensal bacteria (e.g., [7,12,16,17,19,20,23–26,35]). These correlations provide the impetus for experiments to test for direct antagonism between these specific commensals and pathobionts and, when these hypotheses are verified, to uncover the molecular mechanisms involved, as reviewed below.

## What Is Currently Known about Commensal–Pathobiont Interactions in Nasal Microbiota?

Culture-dependent and -independent surveys indicate that non-*diphtheriae Corynebacterium* spp. commonly colonize the pediatric and adult nasal passages [8,11,12,16,19–26,29–34,36,37]. Yet, the function of *Corynebacterium* spp. in these habitats is understudied and remains poorly understood. This is likely because most commensal *Corynebacterium* spp. do not cause disease. Culture-dependent studies indicate that *C*. *accolens*, *C*. *tuberculostearicum*, *C*. *amycolatum*, *C*. *aurimucosum*, *C*. *propinquum*, and *C*. *pseudodiphtheriticum* commonly colonize the adult nose [33,37]. Data on which species colonize the pediatric nose are sparse, but a recent 16S rRNA gene survey of pediatric nasopharyngeal samples detected operational taxonomic units (OTUs) resembling *C*. *accolens* and *C*. *pseudodiphtheriticum*/*C*. *propinquum* [20]. The observed inverse correlation between relative abundances of *Corynebacterium* spp. and *S*. *pneumoniae* in the noses of children under seven years old leads to the hypothesis that antagonism exists between these two groups of bacteria and that *Corynebacterium* spp. might be protective against pneumococcal colonization [23,26]. We observed that in vitro *C*. *accolens*, a lipid-requiring species, releases antipneumococcal free fatty acids from representative human skin surface triacylglycerols; we also identified a primary *C*. *accolens* triacylglycerol lipase [26]. This might represent a mechanism by which *C*. *accolens* antagonizes *S*. *pneumoniae* growth in vivo, thus contributing to colonization resistance against *S*. *pneumoniae*.

An inverse correlation between the genus *Corynebacterium* and *S*. *aureus* is reported in some studies of adult nasal microbiota [15,16,19,22,25], a few of which have examined this at the species level for *Corynebacterium* [16,19]. For example, in a cohort of 40 healthy adults, *S*. *aureus* negatively correlated with higher relative abundance of *C*. *accolens* and positively correlated with *C*. *pseudodiphtheriticum* [16]. In contrast, in a cohort of twelve adults, six with persistent *S*. *aureus* nasal colonization, *C*. *accolens* positively correlated with *S*. *aureus* colonization and in vitro *S*. *aureus* enhanced *C*. *accolens* growth [19]. In the same study, *C*. *pseudodiphtheriticum* negatively correlated with *S*. *aureus* and inhibited *S*. *aureus* growth during in vitro cocultivation [19]. The variation of results between different studies speaks to the potential complexity of *S*. *aureus–Corynebacterium* interactions, including the possibility of strain-level variations, and highlights the need for research to determine the molecular mechanisms involved, which are unlikely to be limited to inhibition.

In considering whether commensal *Corynebacterium* spp. might have a future role in managing nasal microbiota composition, there are precedents for testing commensal *Corynebacterium* spp. as probiotics for eradication of *S*. *aureus* nasal colonization, albeit in small cohorts [15,38]. For example, Uehara and colleagues report that repeatedly implanting an unidentified *Corynebacterium* sp. (Co304) eradicated *S*. *aureus* colonization in 12 of 17 healthy adult carriers [15].

Additional examples of commensal–pathobiont interactions have been described for cutaneous *Propionibacterium* spp., which include *P*. *acnes*, *P*. *avidum*, and *P*. *granulosum*. These are common members of the nostril microbiota in late adolescence and adulthood [7,16,19,21,29–34] and are also detected on nasal mucosal surfaces [19,33,36]. Humans appear to be unusual among mammals in hosting *Propionibacterium* on the skin, and this might relate to the abundance of triacylglycerols in human sebum and skin-surface lipids [39]. *P*. *acnes* is the dominant bacterial inhabitant of the pilosebaceous glands (pores) of skin (Fig 1) [40] and is present on the skin of most adults, at least in developed countries [29,31,34]. (A note of caution: the more recent next-generation sequencing (NGS) studies that rely on the V4 region of the 16S rRNA gene fail to detect the same levels of *Propionibacterium* as prior 454 studies [41], likely because the commonly used 806R primer poorly detects *Propionibacterium*.) Given the ubiquity of *Propionibacterium* on adult human skin surfaces, including the nasal vestibules, several studies have examined potential interactions between cutaneous *Propionibacterium* spp. and *S*. *aureus*. For example, coproporphyrin III (CIII), a diffusible small molecule excreted by nostril- and skin-associated *Propionibacterium* spp., induces *S*. *aureus* aggregation and biofilm formation; this activity is dependent on dose, growth phase, and pH [42]. In other work, Huang and colleagues published several studies that describe effects of *P*. *acnes* on *S*. *aureus* virulence and/or growth in skin wounds [43,44]. Lo et al. showed that *P*. *acnes*-secreted Christie–Atkins–Munch-Petersen (CAMP) factor enhances the hemolytic and cytolytic activity of *S*. *aureus*-secreted beta-hemolysin (sphingomyelinase C) [44]. In a mouse skin infection model, when compared to monoinfection, coinfection of *P*. *acnes* with *S*. *aureus* enhanced *S*. *aureus* virulence in vivo in a manner dependent on active CAMP factor and beta-hemolysin [44]. Shu et al. demonstrated that, when *P*. *acnes* and ^13^C-labeled glycerol are injected into a mouse ear, *P*. *acnes* ferments glycerol, a carbon source available on human skin, to short-chain fatty acids, e.g., propionic acid. These products of *P*. *acnes* glycerol fermentation inhibited growth of USA300 CA-MRSA both in vitro and in vivo in a mouse model of skin wounds [43], although it was unclear how much of this was due to low pH alone. It is likely that these interactions observed on skin surfaces outside of the nasal passages will be applicable to commensal–pathobiont interactions on the skin surfaces of the nasal vestibule.

In addition to the *Corynebacterium–*pathobiont and *Propionibacterium–*pathobiont interactions described above, interactions between *Staphylococcus epidermidis*, a commensal, and *S*. *aureus* have also been investigated. This is driven largely by the hypothesis that, due to their close phylogenetic relationship, these species might compete for a similar niche within the nasal habitat. Because much of this research has been recently reviewed (e.g., [45,46]), it is not covered here.

## What Are Future Research Directions in This Area?

The research discussed here lays the foundation for future testing of harmless nasal bacterial species, e.g., *C*. *accolens*, as potential probiotics. These studies also set the stage for identifying commensal-produced small molecules with the potential for use in managing nasal microbiota composition to promote health. For example, recently developed algorithms that can identify biosynthetic gene clusters whose products synthesize small molecules that might be involved in interspecies interactions within human microbiota promise rapid expansion in this area of research [47,48]. In addition, we expect that microbiota composition studies will continue to lead to the recognition of potentially important, yet previously neglected, commensals, e.g., *Dolosigranulum pigrum*, which is overrepresented in children without *S*. *pneumoniae* nasal colonization and which in older adults appears to be an informative predictor for the lack of *S*. *aureus* colonization [7,17,26]. This review highlights an exciting early stage in the exploration of the molecular mechanisms of interspecies interactions that sculpt nasal microbiota composition and influence pathobiont colonization. Much of this work will also relate directly to the composition of skin microbiota. Because pathobiont colonization is a prerequisite for infection and transmission, a rational approach to prevent infections is to limit or decrease pathobiont abundance and to shift pathobiont behavior towards commensalism using either commensal-derived compounds or commensals as probiotics. We look forward to an increase in research on commensal–pathobiont interactions within the human microbiome and an ever-increasing understanding of the functional significance of our commensal and mutualist bacterial partners.
